# Negative interactions between Toscana virus and *Leishmania infantum* limit coinfection in sand flies

**DOI:** 10.1186/s13071-025-07237-5

**Published:** 2026-02-12

**Authors:** Marketa Stejskalova, Nikola Polanska, Sophie Desloire, Maxime Ratinier, Petr Volf, Magdalena Jancarova

**Affiliations:** 1 Charles University, Prague, Czech Republic; 2 Infections Virales et Pathologie Comparée (IVPC) UMR754, L’Institut national de recherche pour l’agriculture, l’alimentation et l’environnement (INRAE), Universite, l’École Pratique des Hautes Études (EPHE), Université Paris Sciences & Lettres (PSL), Claude Bernard Lyon 1, Lyon, France

**Keywords:** *Leishmania infantum*, Toscana virus, Sand fly, Coinfections, Pathogen–pathogen interactions

## Abstract

**Background:**

Phlebotomine sand flies transmit a wide range of human and veterinary pathogens, including *Leishmania* spp. and Toscana virus (TOSV). Both pathogens co-circulate extensively in the Mediterranean basin and may share hosts and vectors, raising the possibility of mixed infections with epidemiological relevance. While previous studies have suggested interactions between TOSV and *Leishmania* in mammalian hosts and in vitro systems, evidence from natural vectors is still lacking. Understanding these interactions is essential for predicting transmission outcomes in areas of pathogen overlap.

**Methods:**

We investigated coinfection dynamics of TOSV and *Leishmania infantum* in their natural vector, *Phlebotomus tobbi*. Female sand flies were experimentally challenged with both pathogens through blood feeding. We measured infection rates, dissemination rates, and infection intensity levels at days 4 and 8 postinfection (p.i.) and compared the coinfected groups with the control harboring a single infection.

**Results:**

At day 4 (D4) p.i., the coinfection resulted in significant suppression of both pathogens: TOSV infection rates decreased, as did *L. infantum* infection rates. However, neither infection intensity nor viral dissemination showed significant differences between groups. By day 8 (D8) p.i., *L. infantum* maintained a negative effect on TOSV infection, while TOSV did not alter *L. infantum* development. Dissemination and parasite load remained unaffected.

**Conclusions:**

Our findings suggest competitive interactions between TOSV and *L. infantum* in sand flies, providing the first experimental indication of pathogen–pathogen interference within a natural vector. Such competition likely contributes to the rarity of coinfected sand flies in field surveys and highlights the importance of considering vector-level interactions when assessing transmission risks in endemic regions.

**Graphical Abstract:**

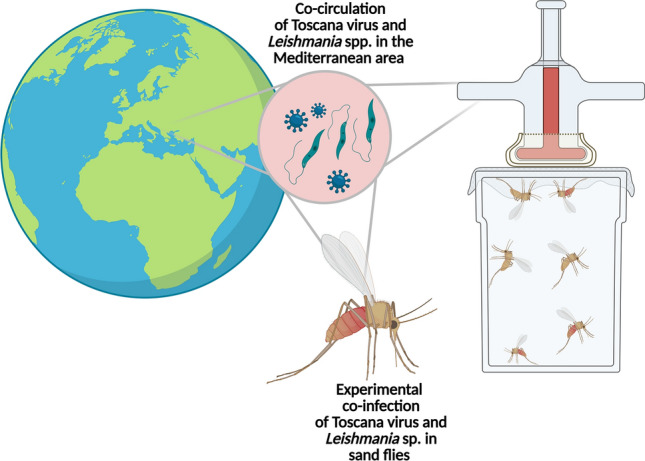

**Supplementary Information:**

The online version contains supplementary material available at 10.1186/s13071-025-07237-5.

## Background

Phlebotomine sand flies (Diptera: Psychodidae) are tiny, nocturnal insects of major veterinary and medical importance, as they are vectors of various pathogens infecting humans as well as domestic and wild animals. Among these pathogens are parasites of the genus *Leishmania*, the causative agents of leishmaniasis. Nevertheless, sand flies also transmit other pathogens, namely bacteria of the genus *Bartonella* and various viruses (reviewed by [1, 2]).

The distribution of sand flies, as well as the range of pathogens they transmit, is currently affected by climatic and environmental changes [3]. Some pathogens have overlapping distributions and may share both vertebrate hosts and arthropod vectors, which can have very important epidemiological consequences [4, 5]. In such cases, mixed infections in hosts or vectors may occur either as: (i) coinfection, when two or more pathogens infect an insect vector simultaneously, or (ii) subsequent infection, which refers to the sequential infection of the vector by different pathogens during successive blood feedings [6]. These mixed infections can lead to three possible outcomes: (i) no interaction [7, 8]; (ii) a facilitative effect, where at least one pathogen profits from this co‐occurrence [9]; or (iii) competition, where one pathogen reduces the fitness of the second one [10–12].

Co-circulating in the Mediterranean region, both transmitted by the same vector—a phlebotomine sand fly—are two important human pathogens: Toscana virus (TOSV; *Phlebovirus toscanense*), a causative agent of central nervous system diseases, and *Leishmania infantum*, the etiological agent of visceral leishmaniasis (VL) (reviewed by [13]). However, their interactions are poorly understood. Bichaud et al. [14] demonstrated by retrospective serological evaluation an epidemiological link between TOSV and *L. infantum* in humans: TOSV‐positive individuals were at higher risk of *Leishmania* infection and vice versa [14]. Similarly, Dincer et al. [15] reported co-circulation and coinfections or subsequent infection with TOSV and *L. infantum* in two dogs in Turkey. However, neither study could determine whether these cases resulted from bites of coinfected sand flies or sequential bites by sand flies with single infections [14, 15]. Given the wide geographic area where these two pathogens co-circulate (reviewed by [13]), the lack of direct evidence of their interactions or the occurrence of coinfections and subsequent infections in vectors remains striking. Research on this subject may have immense epidemiological importance, as studies on animals have shown that phleboviral infection contributes significantly to the promotion of *Leishmania* infection [16–18]. Moreover, Dos Santos et al. [19] suggested that phleboviruses may also benefit from coinfection with *Leishmania* within macrophages.

Importantly, all current evidence comes from vertebrate hosts or cell cultures. To date, no studies have documented coinfections or subsequent infection of sand flies with TOSV and *Leishmania* sp., despite repeated screening in areas where both pathogens co-circulate (e.g., [20–24]. Here, to our knowledge, we report the first study on *Leishmania* and TOSV coinfections in sand flies. Our results suggest competitive interactions between TOSV and *L. infantum* in the natural vector *Phlebotomus tobbi* and provide the first experimental indication of its coinfection interference.

## Methods

### Sand flies

For all experiments, a colony of *Phlebotomus tobbi* (originating from Turkey) was used. This sand fly colony is well established and maintained in the Laboratory of Vector Biology at Charles University, Prague, as described previously [25]. It has tested negative for the presence of phleboviruses, *Leishmania* and *Wolbachia* sp. Previous studies have demonstrated that *Phlebotomus tobbi* is susceptible to *Leishmania infantum* [26] as well as to TOSV [27]. In all experiments females 3–7 days old were used and were maintained at 26 °C with access to 50% sucrose after infection.

### *Leishmania*

*Leishmania infantum* promastigotes (ITOB/TR/2005/CUK3, passage 5) were used and maintained at 23 °C in M199 medium enriched by 10% fetal calf serum (Gibco), 1% Basal Medium Eagle (BME) vitamins (Sigma-Aldrich, Prague, Czech Republic), 2% sterile urine, and 250 μg/mL amikacin (Amikin, Bristol-Myers Squibb, Prague, Czech Republic). Before experimental infection, the parasites were washed by centrifugation (6000 × g for 5 min) and resuspended in saline solution (0.9% NaCl, Braun).

### Cell culture

VeroE6 cells (obtained from Philippe Marianneau, Unité de virologie—ANSES Lyon, France) were used for virus titration by end-point dilution assay (50% tissue culture infectious dose [TCID_50_]). BSR cells (a clone of BHK-21 cells, kindly provided by Karl-Klaus Conzelmann, Ludwig-Maximilians-University Munich, Gene Center, Munich/Germany) [28] and BSR T7/5 CL21 (a clone obtained from the BSR T7/5 cell line, kindly provided by Alain Kohl, Centre for Neglected Tropical Diseases, Liverpool, UK) [29] were used for virus stock production and the reverse genetics method, respectively. All cells were cultivated in Dulbecco’s Modified Eagle medium with high glucose, L-glutamine, sodium pyruvate, and phenol-red (DMEM) supplemented with 10% fetal bovine serum (FBS) and 1% penicillin–streptomycin (P/S), in 75 cm^2^ plastic tissue-culture flasks with filtered screw cap, placed horizontally in a CO_2_ incubator (5% CO_2_, 37 °C).

### Reverse genetics and viral stock production

TOSV MRS2010-4319501 (rgTOSV-B) used in this study was obtained by reverse genetics as described previously [30]. Briefly, BSR T7/5 CL21 cells were transfected by a mixture of three plasmids expressing the antigenome of L, M, and S segments of TOSV-B using lipofectamine 2000 (Invitrogen). After the appearance of the cytopathic effect (CPE), the cell supernatant, named passage 0 (p0), was harvested, clarified by centrifugation, and stored at −80 °C until used to infect VeroE6 for rgTOSV-B virus multiplication. rgTOSV-B stocks for sand fly infections were prepared in BSR cells as previously described [27].

### Experimental infections

Infection experiments were conducted under BSL2 conditions according to the national regulations. In each experiment, three groups of 120–150 *P. tobbi* females were used:

(i) Control TOSV: infection with rgTOSV-B at an infectious dose of 10^6^ plaque forming units (PFU/mL).
(ii) Control *L. infantum*: infection with *L. infantum* at an infectious dose of 10^6^ cells/mL.
(iii) Coinfection: infection with both rgTOSV-B and *L. infantum* at the infectious doses described above.

Since only a few studies have investigated experimental infections with TOSV in sand flies, we based our approach on previous work [27], which demonstrated that an infectious dose of 10^6^ PFU/mL can establish a systemic and disseminated infection. Similar results were reported in *Phlebotomus perniciosus* experimentally infected with TOSV [31].

Briefly, each group of sand fly females was allowed to feed through a chick skin membrane [25] on heat-inactivated ram blood (LabMediaServis s.r.o.) with the appropriate pathogen or their mixtures for 90–120 min. Subsequently, unfed females were separated, while fed ones were dissected on various days postinfection (p.i.): two females were sampled on day 0 p.i. (control of successful infection), and other females were sampled on D4 p.i. and D8 p.i. The head with salivary glands (H) was separated from the rest of the body (B). Before processing, each head with salivary glands was rinsed in a clean drop of sterile physiological saline to remove residual hemolymph. Both samples were manually homogenized with a plastic pestle in 1000 µL crushing medium (DMEM, 4% FBS, amphotericin B, nystatin 100 U/mL, gentamycin 50 g/mL, penicillin–streptomycin 50 g/mL). The resulting homogenate was aliquoted into one 500 µL (used for TCID_50_) and two 250 µL aliquots (used for DNA isolation and backup), and all samples were stored at −80 °C until use.

### Virus detection by end-point dilution assay (TCID_50_)

Ninety-six well plates were filled with 100 µL of DMEM medium supplemented with 4% fetal bovine serum (FBS) and 1% penicillin/streptomycin (P/S). In the first row, 111 µL of each tested sample was added. All samples were tested in quadruplicate. Tenfold serial dilutions of each sample were prepared on a plate. In the next step, 100 µL of VeroE6 cells (4 × 10^4^ cells/mL) were added to each well. Plates were incubated for five days (37 °C, 5% CO_2_).

After incubation, cells from each well were examined under an inverted microscope to evaluate the presence of a virus-induced cytopathic effect (CPE). Wells showing visible CPE were scored as positive; wells without observable changes were considered negative.

Each endpoint dilution assay also included two control conditions: wells containing only uninfected cells without sand fly homogenate and wells containing homogenates from blood-fed females from the *L. infantum* group. Viral titers were calculated using the Reed and Muench method and expressed as the 50% infectious dose of tissue culture per milliliter (TCID_50_/mL) [32].

### Isolation of DNA and preparation of *Leishmania* DNA standard curve

For the detection of *L. infantum* in sand flies, whole DNA was isolated using the HighPure PCR Template Preparation Kit (Roche) from the 250 µL sample aliquot made from the homogenized body. DNA was isolated according to the manufacturer’s protocol and for DNA elution 50 µL of elution buffer was used.

The cell concentration of *L. infantum* culture was counted and diluted to 10^7^ cells/mL, and 1 mL of this culture was used for DNA isolation as described above. In the DNA elution step, 200 µL of elution buffer was used. This DNA was used as stock, and for the standard quantitative polymerase chain reaction (qPCR) curve, six steps of serial dilution (10^6^ − 10^1^ DNA from cells/mL) were prepared fresh before each qPCR run.

### Detection of *Leishmania infantum*

The infection of sand flies by *L. infantum* was tested by qPCR. The primers for kinetoplast DNA were used according to [33] (forward primer 5′-CTTTTCTGGTCCTCCGGGTAGG-3′, reverse primer 5′-CCACCCGGCCCTATTTTACACCAA-3′). Roche LightCycler 480 was used for qPCR with the following program: 98 °C enzyme activation for 3 min, 40 cycles of amplification (98 °C for 10 s, 61 °C for 25 s), melting curve analysis (90 °C), and cooling step (37 °C for 10 min). The 10 µL reaction mixture was prepared as follows: 5 µL LightCycler 480 SYBR Green Master (Roche), 0.25 µL forward primer (10 µM), 0.25 µL reverse primer (10 µM), 1 µL DNA template, 4.5 µL polymerase chain reaction (PCR)-clean water. The final DNA concentration was counted using LightCycler 480 SW (version 1.5.1), and the second derivative maximum method was used for standard curve analysis.

### Statistical analysis and data visualization

The data were analyzed in R (http://cran.r-project.org) [34]. Due to the type of data, the chi-squared test was used for infection rates, the Wilcoxon test for comparison of Toscana virus titers and *Leishmania* quantification, and Fisher’s exact test for Toscana virus dissemination; *P* values < 0.05 were considered statistically significant. The normality of the data distribution was tested with the Shapiro–Wilk test.

## Results

To investigate any effect of TOSV and *L. infantum* coinfection in sand flies, *P. tobbi* females were orally challenged with these two pathogens. We assessed the infection rate (number of pathogen-positive/total exposed sand flies), the dissemination rate (TOSV-infected heads/TOSV-positive bodies), and the intensity of infection (TOSV infectious titer, number of *L. infantum*).

**Fig. 1:**
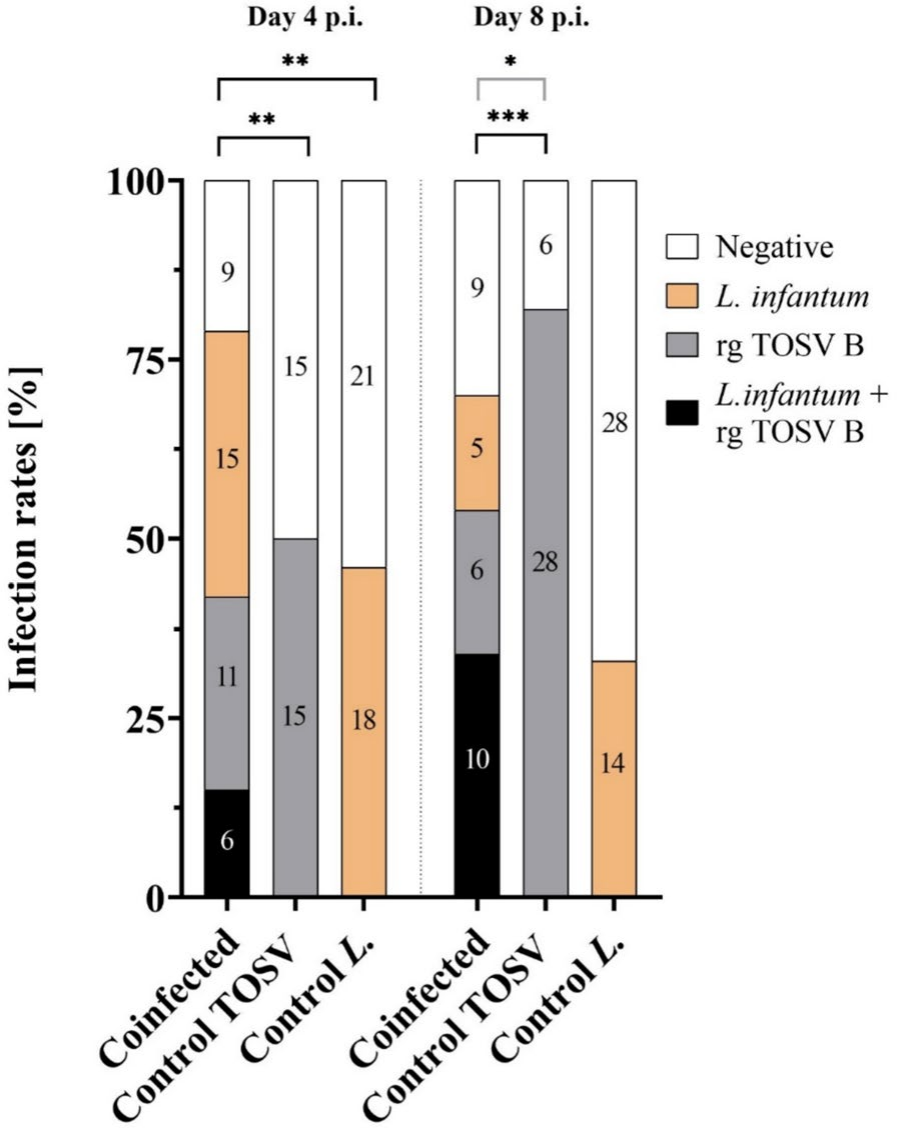
Infection rates in *P. tobbi* females from coinfection with TOSV and *L. infantum* compared with those in control groups at days 4 and 8 p.i. The coinfection group was fed on blood containing both pathogens, *L. infantum* and TOSV, whereas the control groups were fed on blood containing either TOSV-only or *L. infantum*-only. For comparison purposes, the coinfection group was evaluated in three ways: (i) Both pathogens (black): the proportion of females positive for both *L. infantum* and TOSV was compared with both control groups (TOSV-only and *L. infantum*-only). (ii) TOSV-positive (black and grey): all TOSV-positive females in the coinfection group, regardless of *L. infantum* presence, were compared with the TOSV-only control group. (iii) *L. infantum*-positive (black and orange): All *L. infantum*-positive females in the coinfection group, regardless of TOSV presence, were compared with the *L. infantum*-only control group. The numbers indicate the absolute count of positive sand flies

**Fig. 2:**
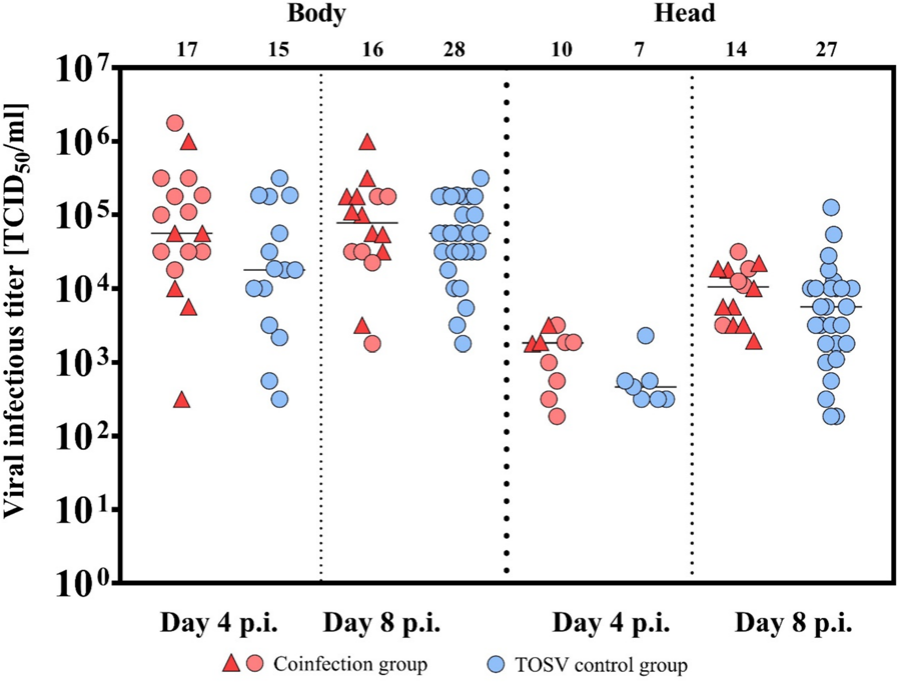
Toscana virus titers in *P. tobbi* infected with TOSV and *L. infantum* at days 4 and 8 p.i. Females in the coinfection group were fed on blood containing both *L. infantum* and TOSV. At days 4 and 8 p.i. Samples positive for TOSV only are indicated with red circles. The red triangles indicate samples in which both *L. infantum* and TOSV were detected. The TOSV control group consisted of females fed on blood containing only TOSV. Positive samples are shown with blue circles. Virus titers are shown separately for bodies and heads, with detection in heads indicating viral dissemination. The numbers above the graph indicate the absolute number of positive samples (body or head). Median values are shown; statistical significance was assessed using the Wilcoxon test

**Fig. 3:**
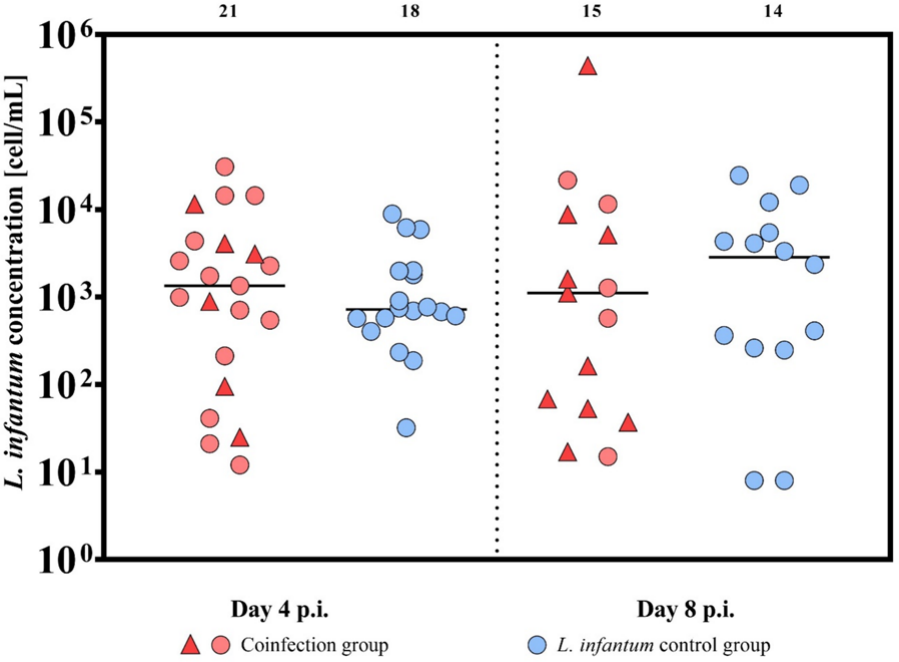
*Leishmania infantum* quantification in *P. tobbi* infected with *L. infantum* and TOSV at days 4 and 8 p.i. The females in the coinfection group were fed on blood containing both *L. infantum* and TOSV and collected on both days 4 and 8 p.i. Red triangles indicate samples in which both *L. infantum* and TOSV were detected. Red circles indicate samples in which only *L. infantum* was detected. The control group consisted of females fed only blood containing *L. infantum*, and positive samples are shown a blue circles. The numbers above the graph indicate the absolute count of positive sand flies. Median values are shown; statistical significance was assessed using the Wilcoxon test

The coinfection group was evaluated in three categories: (i) both pathogens detected (TOSV and *L. infantum* (Figs. 1, 2, 3), (ii) Toscana virus (Figs. 1, 2), or (iii) *L. infantum* (Figs. 1, 3). The infection rates are summarized in Table 1.

**Table 1:** Infection rates of *Phlebotomus tobbi* females experimentally infected with Toscana virus and *Leishmania infantum* on days 4 and 8 p.i.

	Coinfection group			Control groups	
	TOSV + *Leishmania*	TOSV	*Leishmania*	TOSV-only	*Leishmania*-only
Day 4 p.i.					
Positive	6 (14.6%)	17 (41.5%)	21 (51.2%)	15 (50%)	18 (46. 2%)
Negative	35 (85.4%)	24 (58.5%)	20 (48.8%)	15 (50%)	21 (53.8%)
Day 8 p.i.					
Positive	10 (33.3%)	16 (53.3%)	15 (50%)	28 (82.4%)	14 (33.3%)
Negative	20 (66.7%)	14 (46.7%)	15 (50%)	6 (17.6%)	28 (66.7%)

Coinfection groups were compared with single infections (TOSV-only and *Leishmania*-only)

First, we observed a TOSV infection rate of 50% and 82% in the TOSV control group at days 4 and 8 p.i., respectively. Additionally, the *L. infantum* infection rate in the control group was 46% and 33% at days 4 and 8 p.i. Under these conditions, we expected to be able to evaluate both negative and positive interactions between these two pathogens.

When comparing all TOSV-positive females in the coinfection group, regardless of *L. infantum* infection, with the TOSV-only control group, none of the parameters tested were significant at D4 p.i. (infection rate: *χ* ^2^= 0.223, *df* = 1, *P* = 0.637; titer in head: *W* = 50.5, *P *= 0.139; in body: *W* = 171.5, *P *= 0.099). The virus disseminated in 59% of females in the coinfection group and in 46% of females in the control group (*χ*^2^ = 0.111, *df* = 1, *P* = 0.739). However, at D8 p.i., the proportion of TOSV-infected females in the coinfection group was 53% compared with 82% in the TOSV control group, indicating that the presence of *L. infantum* reduced the proportion of TOSV-positive females (*χ* ^2^= 4.969, *df* = 1, *P* = 0.026; Fig. 1). There were no significant differences in the infectious titers and the TOSV dissemination rate between the coinfection and control groups (titer in the head: *W* = 255, *P* = 0.070; body: *W* = 246.5, *P* = 0.587; dissemination rate: 88% in the coinfected group and 97% in the control group, Fisher’s exact test, *P* = 0.285; Fig. 2).

Within the *L. infantum* coinfection group, all *L. infantum*-positive females, regardless of TOSV presence, were compared with the *L. infantum* control group. At days 4 and 8 p.i., neither the infection rate (D4 p.i.: *χ*^2^ = 0.053, *df* = 1, *P* = 0.817; D8 p.i.: *χ*^2^ = 1.387, *df* = 1, *P* = 0.2389; Fig. 1) nor the infection intensity (D4 p.i.: *W* = 211, *P* = 0.545; D8 p.i.: *W* = 95, *P* = 0.683; Fig. 3) differed significantly between the groups.

One limitation of the analysis is that, in some individuals in the coinfected group, both pathogens were not detected. Therefore, we also compared coinfected individuals (TOSV and *L. infantum* positive) with both control groups. We observed a significantly reduced number of positive females in the coinfected group compared with the TOSV and the *L. infantum* control groups (*χ*^2^ = 8.774, *df* = 1, *P* = 0.003 and *χ*^2^ = 8.015, *df* = 1, *P* = 0.0046, respectively) in the early phase of infection (day 4 p.i.) (Fig. 1). However, neither the intensities of infection of both pathogens nor the TOSV dissemination rate differed significantly between the coinfected and control groups (*L. infantum* intensity of infection: *W* = 60, *P* = 0.721, Fig. 3; TOSV infectious titers in the head: *W* = 19, *P* = 0.064; in body: *W* = 36, *P* = 0.308; dissemination rate: 50% in the coinfected group and 46% in the control group, Fisher’s exact test, *P* = 1; Fig. 2).

In the later phase of infection (day 8 p.i.), the infection rate of both *L. infantum* and TOSV was significantly lower compared with the TOSV control group (*χ*^2^ = 13.91, *df* = 1, *P* = 0.0002), while no significant differences were observed when compared with the *L. infantum* control group (*χ*^2^ = 0, *df* = 1, *P* = 1; Fig. 1). Similarly, there were no significant differences in the dissemination rate (91% in the coinfected group and 97% in the control group, Fisher’s exact test, *P* = 0.476), the intensity of *L. infantum* infection (W = 57, *P* = 0.472; Fig. 2), or the intensity of the TOSV infection (TOSV infectious titer in the head: *W* = 150.5, *P* = 0.294; in body: *W* = 92, *P* = 0.541; Fig. 2) between the coinfected and control groups.

Altogether, at a later time postinfection (day 8 p.i.), we observed a negative interaction of *L. infantum* on the TOSV infection rate, whereas TOSV infection does not significantly modulate the *L. infantum* infection rate regardless of how we define the coinfected group. Interestingly, the coinfection of *P. tobbi* by both TOSV and *L. infantum* has no impact on their intensity of infection and the TOSV dissemination rate. Also, at D4 we could see the mutual negative effect of *Leishmania* and TOSV in *P. tobbi*.

Detailed information on viral titers, Cp (crossing point) values, and *L. infantum* concentration is shown in the Additional file 1: Table S1.

## Discussion

This study focuses on the coinfection of TOSV and *L. infantum* through blood feeding in their natural vector *P. tobbi*. Our results indicate that these two pathogens seem to compete each other. During the early phase of infection, they exhibit negative interactions, leading to a significant reduction in the number of coinfected sand fly females in the coinfection group (those females where both pathogens were detected). However, in the later phase, only *Leishmania* exerts a negative effect on TOSV, again resulting in a lower number of coinfected females, and this impact was confirmed regardless of how the data were analyzed.

Relatively high heterogeneity observed across experimental replicates is an inherent characteristic of sand-fly infections, which are biologically variable due to differences in individual vector susceptibility. Variability is not due to the virus, as the virus for all replicates originates from the same stock; rather, it is due to sand flies and their individual vector susceptibility, which might be affected by diversity of physiological/immune status of the fly and the midgut microbiome [35]. Such variability has also been documented in the literature on phlebotomine experimental infections, where individual flies showed differences in infection rates and parasite loads even under controlled laboratory conditions [26, 36–38]. We expect that similar variability occurs in field conditions, where environmental and ecological factors further contribute to heterogeneity in infection outcomes.

The negative interaction between TOSV and *Leishmania* may explain why coinfected sand flies are rarely observed in field studies in areas where both pathogens circulate [20, 22, 39]. To date, there is only one record of natural coinfection with TOSV and *Leishmania* in sand flies, one pool of 20 *P. tobbi* females collected in Northern Cyprus [21]. However, it remains unclear whether the coinfection occurred in a single individual or in two individuals within the same pool.

The precise mechanism underlying this negative interaction remains unknown. A possible explanation is the role of the sand fly’s immune system, but current information on this topic is very limited. In insects, viral infection typically triggers antiviral immune responses through pathways such as Toll, Imd, and JAK–STAT, with RNA interference playing a crucial role (reviewed by [35]). In sand flies specifically, the activity of the exogenous small interfering RNA (exo-siRNA) pathway against TOSV has been confirmed in a sand fly-derived cell line [40]. In the case of *Leishmania* infection in sand flies, transcriptomic studies have reported differential expression of several immune-related genes, including components of the Toll, Imd, and JNK pathways, as well as molecules related to oxidative stress, such as antioxidants that control reactive oxygen species (ROS) levels (reviewed by [35]). The midgut microbiome also participates in these relationships [41], resulting in a very complex interaction that requires further study.

So far, only one experimental study has examined a virus–*Leishmania* coinfection in sand flies. Chronic infection of *Phlebotomus papatasi* by cytoplasmic polyhedrosis virus (CPVs) leads to structural damage of the midgut epithelium and the peritrophic matrix, rendering females refractory to *Leishmania major* infection [42]. In mosquitoes, a more extensively studied group, information on virus–protozoa coinfection is also limited. In *Anopheles gambiae*, intrathoracic infection with O’nyong’nyong virus (ONNV) followed by a blood meal infected with *Plasmodium berghei* reduced the number of melanized ookinetes, as ONNV upregulated negative regulators of the melanization cascade [43]. However, these results are not directly comparable, as they involve subsequent infection and rely on intrathoracic injection, a method that ensures consistent infectious doses and higher infection rates, but bypasses key barriers such as the peritrophic matrix and the midgut [44].

More recently, studies have been published on the coinfection with phleboviruses and *Leishmania* in mammalian hosts or in vitro cell cultures [16–18]. Rossi et al. [16] reported that the coinfection of TOSV and *Leishmania guyanensis* in a murine model increased parasite burden and lesion size. Similarly, other combinations of *Leishmania* and phlebovirus have been shown to exacerbate *Leishmania* infection [17, 18].

Our findings highlight the epidemiological importance of investigating the coinfection and subsequent infection with *TOSV* and *Leishmania* in phlebotomine sand flies. Although co-circulation of these two pathogens in the same areas has been repeatedly reported (e.g., [13, 22, 45]), this is the first study to investigate their interaction through laboratory experiment. Competitive interactions between Toscana virus and *Leishmania infantum* may reduce the probability of simultaneous transmission by a single sand fly and influence which pathogen predominates in each area or period. Such effects could shape the overall transmission dynamics and maintenance of both pathogens in nature. We suggest that both pathogens interact and negatively influence each other in their natural vector, a finding that may have far-reaching implications for the transmission and spread of both pathogens and the diseases they cause. Nevertheless, considering the relatively low infection rates of hosts by either pathogen, the optimal approach would be to first feed sand flies with *Leishmania*, followed by a second bloodmeal containing the virus (or vice versa), as this scenario is more likely to occur in nature. We advocate for undertaking these extremely challenging experiments in future studies.

## Conclusions

Our findings highlight the epidemiological importance of investigating the coinfection and subsequent infection with TOSV and Leishmania in phlebotomine sand flies. Although co-circulation of these two pathogens in the same areas has been repeatedly reported (e.g., [13, 22, 45]), this is the first study to investigate their interaction through laboratory experiment. Competitive interactions between Toscana virus and Leishmania infantum may reduce the probability of simultaneous transmission by a single sand fly and influence which pathogen predominates in each area or period. Such effects could shape the overall transmission dynamics and maintenance of both pathogens in nature. We suggest that both pathogens interact and negatively influence each other in their natural vector, a finding that may have far-reaching implications for the transmission and spread of both pathogens and the diseases they cause. Nevertheless, considering the relatively low infection rates of hosts by either pathogen, the optimal approach would be to first feed sand flies with Leishmania, followed by a second bloodmeal containing the virus (or vice versa), as this scenario is more likely to occur in nature. We advocate for undertaking these extremely challenging experiments in future studies.

## Supplementary Information

Additional file 1.

## Data Availability

All data generated or analyzed during this study are included in this published article.
